# Inhibition effect of flavophospholipol on conjugative transfer of the extended-spectrum β-lactamase and *vanA* genes

**DOI:** 10.1038/s41429-018-0113-4

**Published:** 2018-10-25

**Authors:** Hayami Kudo, Masaru Usui, Wataru Nagafuji, Kentaro Oka, Motomichi Takahashi, Hiroyuki Yamaguchi, Yutaka Tamura

**Affiliations:** 1 Laboratory of Food Microbiology and Food Safety, Department of Health and Environmental Sciences, School of Veterinary Medicine, Rakuno Gakuen University, Ebetsu, Hokkaido 069-8501 Japan; 2 Tokyo R&D Center, Miyarisan Pharmaceutical Co., Ltd., Saitama-shi, Saitama 331-0804 Japan; 3 Department of Medical Laboratory Science, Faculty of Health Sciences, Hokkaido University Graduate School of Health Sciences, Sapporo, Hokkaido 060-0812 Japan

**Keywords:** Antimicrobial resistance, Antibiotics

## Abstract

Flavophospholipol (FPL) is an antimicrobial feed additive that has been approved for use in livestock animals and has the potential to decrease horizontal dissemination of antimicrobial resistance genes. Since previous studies showed that FPL has an inhibitory effect on plasmid transfer, in vitro experiments have proven the efficacy of FPL in reducing the conjugative transfer of plasmids encoding the extended-spectrum β-lactamase (ESBL) and *vanA* genes. These are among the most important antimicrobial resistance loci known. ESBL-producing *Escherichia coli* and vancomycin-resistant *Enterococcus faecalis* (VRE) were exposed to several concentrations of FPL, and transfer frequency and plasmid curing activity were determined. FPL inhibited the conjugative transfer of plasmids harboring ESBL and *vanA* genes in a concentration-dependent manner in all strains. Further transfer experiments revealed that FPL could decrease or increase transfer frequency depending on plasmid type when transfer frequency was at low levels. The plasmid curing activity of FPL was also observed in ESBL-producing *E. coli* in a concentration-dependent manner, suggesting that they partially contribute to the inhibition of conjugative transfer. These results suggest that the use of FPL as a feed additive might decrease the dissemination of ESBL and *vanA* genes among livestock animals.

## Introduction

Antimicrobials are used in livestock animals to treat bacterial infection. In addition, some antimicrobials are used as feed additives to improve the quality of products and promote growth [1]. However, inappropriate use of antimicrobials can provide an opportunity for antimicrobial resistance, which may spread between livestock animals and humans via food chains or through the environment, increasing the risk of severe infections [2]. Some antimicrobial resistance genes are carried on plasmids and the ease of conjugative transfer of those plasmids allows rapid spread of antimicrobial resistance [3].

The extended-spectrum β-lactamase genes (ESBLs) and some vancomycin (VCM) resistance genes, such as *vanA* and *vanB*, are carried on plasmids and are some of the most intensely researched antimicrobial resistance genes in the world. The plasmid-mediated ESBL genes, which can confer resistance to third-generation cephalosporins, have been observed in isolates derived from livestock animals [4–7]. The third-generation cephalosporins are clinically important antimicrobials due to their little reported side effects and broad-spectrum activity [6]. The *vanA* gene cluster is located on Tn*1546*, which often resides on a plasmid in vancomycin-resistant enterococci (VRE) [8]. VRE possess a high-level resistance to VCM. Moreover, most VRE are resistant to both β-lactam and aminoglycoside antibiotics [8]. VCM is also clinically important as it is recommended for the treatment of infections caused by *Clostridium difficile* and MRSA [9, 10]. Concerningly, the persistence of VRE carrying the *vanA* gene was observed in livestock animals 10 years or more after avoparcin (related to VCM) was banned in some European countries [11, 12]. Thus, efficient countermeasures to prevent the spread and maintenance of plasmid-mediated ESBL and *vanA* genes among livestock animals are required.

Flavophospholipol (FPL), also known as flavomycin, moenomycin, and bambermycin, is an antimicrobial that is produced by *Streptomyces* spp. and used as a feed additive for poultry and piglets in some countries including Japan, but its use is limited in the EU [1]. It is reported that FPL inhibits glycosyltransferase, an enzyme involved in cell wall synthesis. In addition to this mode of action, FPL has showed an inhibitory effect on plasmid transfer [13, 14]. In our knowledge, few studies have tested the inhibitory effect of FPL on a wide range of plasmids belonging to different incompatibility groups. To clarify the inhibitory effect of FPL on conjugational transfer of ESBL and *vanA* genes, we exposed ESBL-producing *E. coli* and VRE, which contains several types of plasmids, to FPL, and determined transfer frequency and plasmid curing activity.

## Materials and methods

### Strains

To determine the inhibitory effect of FPL on conjugative transfer of highly transmissible plasmids, we selected strains for experiments of conjugative transfer with FPL. In the first round transfer experiment, three donor strains of ESBL-producing *E. coli* previously isolated from bovine feces at Rakuno Gakuen University: Dc1 (133), Dc2 (TC13-1), and Dc3 (TC7-1), were used [15, 16]. Dc1, Dc2, and Dc3 contain self-transmissible plasmids carrying the β-lactamase genes (*bla*_CTX-M-15_, *bla*_CTX-M-14_, and *bla*_CTX-M-2_, respectively). All these donor strains were resistant to ampicillin (ABPC), cefazolin (CEZ), and cefpodoxime (CPDX). The incompatibility groups of the β-lactamase gene encoding plasmids of three donor strains are FIB, I1-Iγ, and N, respectively. *E. coli* ML4909 (Rc), a rifampicin (RIF) -resistant derivative of *E. coli* K-12, was used as the recipient strain [17]. The transconjugants (TC) of the first round transfer were used as donor strains to perform a second round transfer experiment (TC obtained from Dc1, Dc2, and Dc3 were named Tc1, Tc2, and Tc3, respectively). *E. coli* DH5α, a nalidixic-acid (NA) resistant derivative of *E. coli* K-12, was used as the recipient strain in the second round transfer experiment.

Two donor VRE strains V1300 (Df1) and V1355 (Df2) isolated from human feces at the University of Occupational and Environmental Health, Japan were used. Df1 and Df2 contain transmissible plasmids positive for *vanA*, the gene that confers resistance to VCM. These plasmids from Df1 and Df2 belong to untypeable incompatibility groups. Rifampicin-resistant *E. faecalis* FA2-2 (Rf) was used as the recipient strain [18].

### Antimicrobial susceptibility testing

The antimicrobial susceptibilities to FPL (BIOVET, Saint-Hyacinthe, USA), reference substance and purified by HPLC, were determined by using agar dilution methods according to the recommendation of the Clinical and Laboratory Standards Institute (CLSI) [19]. The following ATCC strains were used as controls for susceptibility testing: *Staphylococcus aureus* ATCC29213, *E. faecalis* ATCC29212, *E. coli* ATCC25922, and *Pseudomonas aeruginosa* ATCC27853.

### Growth inhibition testing

Each donor and recipient strain was grown overnight at 37 °C in 1 ml of Tryptic Soy Broth (TSB) (Bacto^TM^, New Jersey, USA). 50 μl of the growth culture was added to 1 ml of TSB containing FPL (0, 2, 4, 8, 16, 32, or 64 μg/ml for *E. coli* and 0, 0.016, 0.031, 0.063, 0.125, 0.250, or 0.500 μg/ml for *E. faecalis*) and incubated overnight at 37 °C. 0.1 ml of ten-fold serial dilutions of overnight culture were spread on Tryptic Soy Agar (TSA) (Bacto^TM^) plates containing CEZ (16 μg/ml) for *E. coli* donor strains, VCM (8 μg/ml) for *E. faecalis* donor strains, and RIF (25 or 50 μg/ml) for *E. coli* and *E. faecalis* recipient strains, respectively and incubated overnight at 37 °C before colony counting. Statistical analysis was performed by analysis of one-way ANOVA with Tukey–Kramer test to assess growth inhibition testing.

### First round conjugative transfer with FPL

The broth mating method was used to determine the inhibitory effect of FPL on conjugative transfer of *E. coli* [13, 20]. Briefly, the donor strain was grown overnight at 37 °C in 1 ml of TSB. 50 μl of overnight culture was added to 1 ml of TSB containing 0, 2, 4, 8, 16, 32, or 64 μg/ml FPL. The recipient strain was grown overnight at 37 °C in 1 ml of TSB. 0.1 ml of the donor culture and 0.1 ml of the recipient culture were added to 0.8 ml of TSB containing 0, 2, 4, 8, 16, 32, or 64 μg/ml FPL. This mixture was incubated overnight at 37 °C. 0.1 ml of ten-fold serial dilutions of overnight culture were spread on TSA plates containing selective antibiotics (transconjugant-selective plates contained 16 μg/ml CEZ and 50 μg/ml RIF) and incubated overnight at 37 °C, then colonies were counted.

Because *E. faecalis* used in this study contained transmissible plasmids with molecular size less than 30 kb which were not able to transfer between donor and recipient strains using the broth mating method, the filter mating method was used [14, 20]. Briefly, the donor strain was grown overnight at 37 °C in 10 ml of TSB. 1 ml of overnight culture was added to 5 ml of TSB containing 0, 0.016, 0.031, 0.063, 0.125, 0.250, or 0.500 μg/ml FPL. The recipient strain was grown overnight at 37 °C in 20 ml of TSB. 2.5 ml of the donor culture and 2.5 ml of the recipient culture were then mixed. In the filtration step, 5 ml of mixture was collected on a membrane filter (ADVANTEC, Tokyo, Japan; pore size 0.22 μm). The filters were placed on TSA containing FPL (concentrations as described above) and incubated overnight at 37 °C. The filters were transferred to a centrifuge tube containing 0.5 ml of sodium chloride solution, and washed 3 times. 0.1 ml of ten-fold serial dilutions of this solution were spread on TSA plates containing selective antibiotics (transconjugant-selective plates contained 8 μg/ml VCM and 25 μg/ml RIF) and incubated overnight at 37 °C, then colonies were counted.

Transfer of the plasmid in transconjugants (TC) was verified by PCR analysis and plasmid profiles [21]. DNA was extracted from transconjugants by using InstaGene^TM^ Matrix (Bio-Rad Laboratories, California, USA), according to the manufacturer’s instructions. The PCR analysis, which allows detection of β-lactamase genes and the *vanA* gene, was performed as described previously [22, 23]. The conjugation study was performed three times and the transfer frequency was calculated by the number of transconjugants (cfu/ml) per the number of donors (cfu/ml).

### Second round conjugative transfer with FPL

Using the same method as the first round conjugative transfer experiment, the second round conjugative transfer experiment was performed to determine whether the inhibitory effect of FPL on transfer frequency changed depending on plasmid type. The transconjugants of the first round conjugative transfer experiment were used as the donor cells; transconjugants harboring plasmids of Dc1, Dc2, and Dc3 were renamed Tc1, Tc2, and Tc3, respectively. A nalidixic-acid (NA) resistant *E. coli* DH5α was used as the recipient strain. The transconjugants were selected by 16 μg/ml CEZ and 32 μg/ml NA. As the plasmids of VRE belong to untypeable incompatibility groups, these were not tested.

### Plasmid curing experiment

The plasmid curing of *E. coli* and *E. faecalis* donor strains was performed as described previously [24]. In brief, each donor strain was grown overnight at 37 °C in 1 ml of TSB. 50 μl of the growth culture was added to 1 ml of TSB containing FPL (0, 2, 4, 8, 16, 32, or 64 μg/ml for *E. coli* and 0, 0.016, 0.031, 0.063, 0.125, 0.250, or 0.500 μg/ml for *E. faecalis*) and 100 μg/ml EtBr was used as positive control [25]. 0.1 ml of ten-fold serial dilutions of overnight culture were spread on TSA plates and incubated overnight at 37 °C. The isolated colonies were then replica plated on TSA plates and TSA plates containing CEZ (16 μg/ml) for *E. coli* or VCM (8 μg/ml) for *E. faecalis* using the Replica-Plating Tool (Scienceware,Bel-Art Products, USA) and incubated overnight at 37 °C before colony counting.

The colonies that failed to grow on TSA plates containing selective antibiotics were considered as putative cured isolates. The loss of plasmid in the cured isolates was confirmed by plasmid profile [21]. The percentage of curing efficiency was expressed as number of colonies with cured phenotype per all counted colonies on TSA plates. The experiments were repeated four times. To assess the relationship between curing efficiency and dose of FPL, single regression analysis was performed.

## Results

### Antimicrobial susceptibility testing

The minimum inhibitory concentrations (MICs) for FPL for all *E. coli* donor and recipient strains were 128 μg/ml, and those for all *E. faecalis* donor and recipient strains were 1 μg/ml.

### Growth inhibition testing

FPL treatment significantly inhibited all *E. coli* growth at 32 and 64 μg/ml (Table 1). Moreover, FPL significantly inhibited *E. faecalis* Df1 and Df2 growth at 0.125, 0.25, and 0.5 μg/ml and inhibited *E. faecalis* Rf growth at 0.5 μg/ml (Table 1).

**Table 1:** The growth inhibition effect of different concentrations of FPL on *E. coli* and *E. faecalis* (log10 CFU/ml)

	FPL (µg/ml)													
Strains		0	0.016	0.031	0.063	0.125	0.25	0.5	2	4	8	16	32	64
*E. coli*	Dc1	9.22±0.02^a^	–	–	–	–	–	–	9.23 ± 0.03^a^	9.19 ± 0.02^a^	9.13 ± 0.05^a^	9.16 ± 0.08^a^	8.78 ± 0.19^b^	8.40 ± 0.09^c^
	Dc2	9.26±0.03^a^	–	–	–	–	–	–	9.36 ± 0.13^a^	9.24 ± 0.07^a^	9.20 ± 0.01^a^	9.11 ± 0.06^a^	8.71 ± 0.18^b^	8.68 ± 0.04^b^
	Dc3	9.44±0.18^a^	–	–	–	–	–	–	9.34 ± 0.13^a^	9.30 ± 0.02^a^	9.33 ± 0.02^a^	9.21 ± 0.06^ab^	8.97 ± 0.10^b^	8.27 ± 0.06^c^
	Rc	8.91±0.05^a^	–	–	–	–	–	–	8.91 ± 0.08^a^	8.86 ± 0.13^a^	8.68 ± 0.31^a^	8.61 ± 0.21^a^	7.44 ± 0.53^b^	5.19 ± 0.12^c^
*E. faecalis*	Df1	9.06 ± 0.04^a^	8.99 ± 0.03^a^	9.02 ± 0.05^a^	8.98±0.02^a^	8.53 ± 0.03^b^	8.32 ± 0.02^c^	7.87 ± 0.06^d^	–	–	–	–	–	–
	Df2	8.83 ± 0.12^a^	8.72 ± 0.19^a^	8.75 ± 0.05^a^	8.66±0.02^a^	8.37 ± 0.10^b^	8.02 ± 0.04^c^	7.53 ± 0.09^d^	–	–	–	–	–	–
	Rf	9.23 ± 0.04^a^	9.16 ± 0.15^a^	9.20 ± 0.02^a^	9.25±0.10^a^	9.19 ± 0.01^a^	8.96 ± 0.02^a^	8.59 ± 0.23^b^	–	–	–	–	–	–

Data are presented means ± S.E.

Means followed by the same letters in a column do not significantly differ from each other using the Tukey–Kramer test at *p* = 0.05

― indicates not tested

### Inhibition of conjugation transfer

**Fig. 1:**
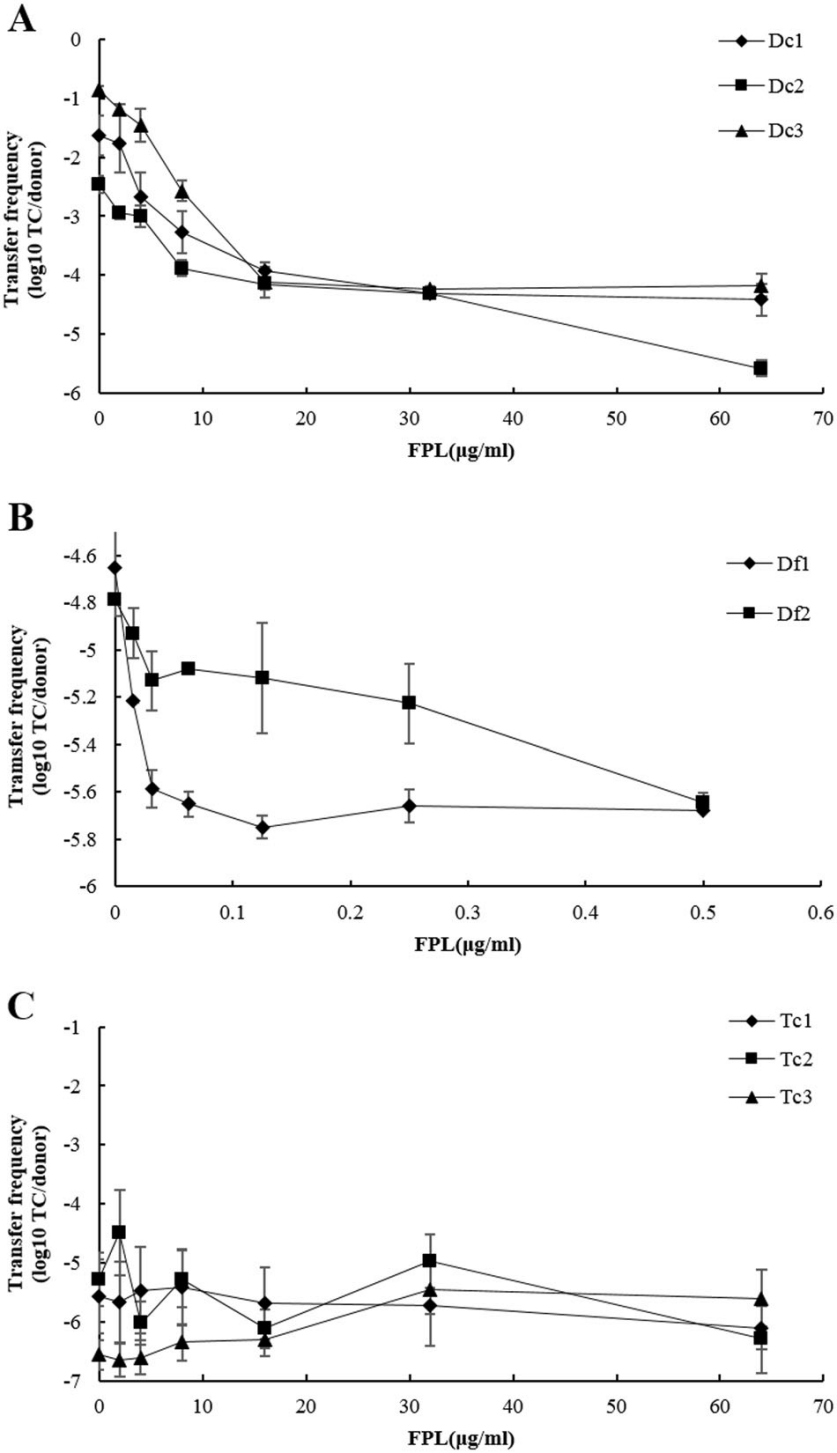
**a** Effect of different concentrations of FPL on the first round conjugative transfer frequency in *E. coli* donor strains; Filled diamonds, filled squares and filled triangles were Dc1, Dc2, and Dc3, respectively. **b** Effect of different concentrations of FPL on the transfer frequency in *E. faecalis* donor strains; Filled diamonds and filled squares were Df1 and Df2, respectively. **c** Effect of different concentrations of FPL on the second round conjugative transfer frequency in *E. coli* donor strains; Filled diamonds, filled squares, and filled triangles were Tc1, Tc2, and Tc3, respectively. Data are presented as means ± SEM

Untreated control transfer frequencies (TC/donor) for *E. coli* donor stains Dc1, Dc2, and Dc3 were 2.3 × 10^−2^, 3.4 × 10^−3^, and 1.4 × 10^−1^, respectively and that for *E. faecalis* donor stains Df1 and Df2 were 2.2 × 10^−5^ and 1.6 × 10^−5^, respectively. For all *E. coli* and *E. faecalis* strains, exposure to FPL significantly decreased the transfer frequency in a concentration-dependent manner (Fig. 1a, b). Approximately 1.4–3.0 fold reduction in transfer frequency was observed when *E. coli* was treated with 2 µg/ml FPL. Similarly, the plasmid transfer in *E. faecalis* was inhibited up to 7.2–10.7 fold in the presence of 0.5 µg/ml FPL.

In the second round conjugative transfer experiment when exposed to 64 µg/ml FPL, the transfer frequency of Tc1 and Tc2 harboring Inc FIB and I1-Iγ type plasmids was decreased compared with control (Fig. 1c). In contrast, the transfer frequency of Tc3, which contained the Inc N type plasmid, was relatively increased by FPL. However, this latter effect did not achieve statistical significance (Fig. 1c).

### Plasmid curing experiment

**Fig. 2:**
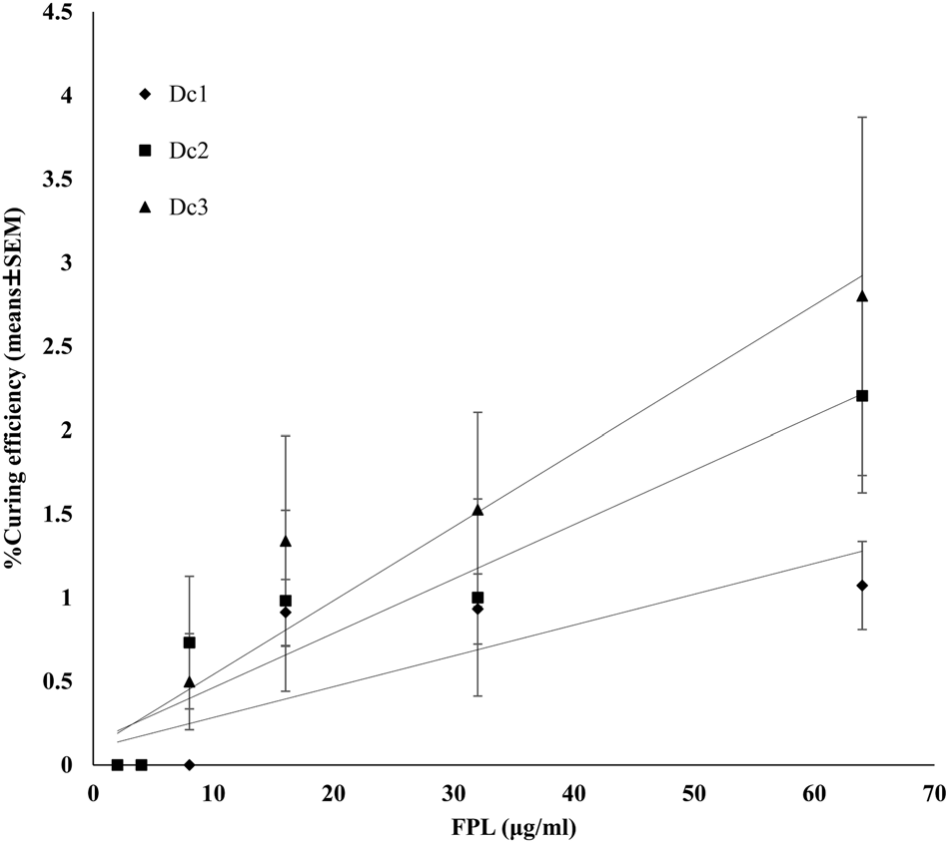
Single linear regression analysis of the relationship between % curing efficiencies and different concentrations of FPL. Solid lines represent each linear line for dose-depend increased of curing efficiencies. Filled diamonds, filled squares, and filled triangles were Dc1, Dc2, and Dc3, respectively. Data are presented as means ± SEM

Plasmid curing activity was observed in Dc1 at 16, 32, and 64 μg/ml FPL treatment and in Dc2 and Dc3 at 8, 16, 32, and 64 μg/ml (Fig. 2). Linear regression analysis revealed that concentration-dependent plasmid-curing activities were significantly observed in all *E. coli* donor strains (Table 2). EtBr was able to cure plasmids successfully at 100 μg/ml, and the curing efficiencies for Dc1, Dc2, and Dc3 were 1.3 ± 0.3%, 1.6 ± 0.4%, and 1.3 ± 0.6%, respectively. Plasmid curing activities of 64 μg/ml FPL against Dc1, Dc2, and Dc3 of *E. coli* were 1.1 ± 0.3%, 2.2 ± 0.6, and 2.8 ± 1.1%, respectively, and were almost the same or relatively higher than those of 100 μg/ml EtBr. No plasmid curing activity was observed when *E. coli* was exposed to low concentrations of FPL (2 and 4 μg/ml). Despite the fact that FPL induced plasmid curing in *E. coli*, no plasmid curing activity was induced in *E. faecalis* at any concentration (data not shown).

**Table 2:** Regression valuables of curing efficiency and different concentrations of FPL treatment

Strains	Slope	*P*-value	95% Confidence interval
Dc1	0.02	0.0004334	0.0096 to 0.0273
Dc2	0.03	0.0006043	0.0163 to 0.0486
Dc3	0.04	0.0002412	0.0232 to 0.0650

## Discussion

In this study, the inhibitory effect of FPL on conjugative transfer was observed in a concentration-dependent manner for all ESBL-producing *E. coli* and VRE strains. Additionally, this reduction of transfer frequency was observed at lower concentrations than those used in the growth inhibition test. The conjugative pilus synthesized by the donor strain is essential for conjugative transfer in Gram-negative bacteria such as *E. coli* [26]. Unlike *E. coli*, Gram-positive bacteria including *E. faecalis* do not use pili and the conjugation system is instead dependent on pheromone-induced conjugation [27]. These results suggest that the inhibition mechanism in Gram-negative and positive bacteria by FPL is not dependent on the pilus.

Approximately, 1.4–3.0 fold reduction in transfer frequency was observed when *E. coli* harboring ESBL genes were treated with 2 µg/ml FPL (the transfer frequencies of untreated controls were −2.46~ −0.86_log10_ TC/donor). In the previous study [13], exposure to 2 µg/ml FPL completely inhibited the conjugative transfer of *E. coli* harboring antibiotic-resistance plasmids (untreated control transfer frequency was at 8.1 × 10^−4^). The difference in transfer frequency between the previous reports and our results may be caused by strain differences or differences in the plasmids used in conjugation experiments. In fact, there was a significant difference even between the transfer frequencies of the three donor strains of *E. coli* when exposed to low concentrations of FPL in this study.

George et al. reported that the inhibitory effect of FPL on conjugative transfer depended on plasmid type [28]. However, in the first round transfer experiments, FPL significantly inhibited conjugative transfer of all plasmids belonging to distinct Inc groups. To confirm whether the inhibitory effects of FPL were really dependent on the type of plasmid, we performed a second round transfer experiment using the same type of donor strains (harboring the plasmids belonging to several Inc groups). We found that FPL decreased the transfer frequencies of Inc FIB and I1-Iγ type plasmids, but increased that of the Inc N type plasmid. However, untreated control transfer frequencies of the second round transfer experiment were significantly lower than those of the first round transfer experiment. These results suggest that FPL decreased or increased transfer frequency depending on plasmid type when transfer frequency was at low levels, whereas it indiscriminately reduced the conjugative transfer of all plasmid types when transfer frequency was high.

The plasmid transfer frequency in *E. faecalis* was inhibited up to 7.2–10.7 fold in the presence of 0.5 µg/ml FPL, which is consistent with the previous study [14].

FPL is approved for use as a feed additive in broilers and pigs at 1–5 g/ton and 2–10 g/ton, respectively [29]. Although FPL may tend to be diluted or accumulated in gut depending on intestinal environment, most FPL is not absorbed in the gut and is excreted in feces [30]. Therefore residual FPL in the gut was considered to be the same as the approved concentration. For all *E. coli* and *E. faecalis* strains, inhibition of conjugational transfer was observed at 1–10 µg/ml FPL. However, approved concentrations of FPL are below the MIC level of *E. coli* (128 µg/ml) and above the MIC level of *E. faecalis* (1 µg/ml), suggesting that the above-approved concentration would have bactericidal actions for Gram-positive bacteria such as *E. faecalis* and sufficiently inhibit conjugative transfer of ESBL and *vanA* gene encoding plasmids. Resistance gene transfer is most likely to occur in feces [31] and FPL may act most effectively at preventing transfer in this medium. In fact, Bogaard et al. reported that FPL effectively suppressed the increase and dissemination of multi-resistant *E. coli* in the intestinal flora of pigs but increased FPL-resistant *E. faecalis* [32]. By contrast, there was no significant difference in the number of transconjugants between untreated control and FPL treated chickens [13].

FPL exhibited plasmid curing activity in *E. coli* harboring a plasmid carrying ESBL genes in a concentration-dependent manner significantly. The plasmid curing activities of a high concentration of FPL were almost the same as those of EtBr, which is well known as a plasmid curing agent [25]. These results suggest that plasmid-curing likely contributes partially to the inhibition of conjugational transfer at high concentrations. By contrast, FPL had no plasmid-curing effect in *E. faecalis*. However, Keyhani et al. reported that no plasmid curing activity was observed when *E. faecalis* was exposed to either acridine orange or SDS, other known plasmid curing agents in *E. coli* [33], suggesting that species differences may explain this result.

In conclusion, our results suggest that the approved concentration of FPL for use as a feed additive in livestock animals would inhibit conjugational transfer of ESBL and *vanA* genes in the gut or in animal feces. The use of FPL as a feed additive may decrease the risk of dissemination of plasmid-mediated resistance genes from animal to human via the food chain.
